# *Ganoderma lucidum* Glycoprotein Microemulsion: Improved Transdermal Delivery and Protective Efficacy in UV-Induced Cell and Animal Models

**DOI:** 10.3390/molecules30224489

**Published:** 2025-11-20

**Authors:** Ye Jin, Xushuang Jia, Dongmei Fan, Xuyan Zhou, Xiao Tan, Da Liu, Ning Cui, Jiawei Wen

**Affiliations:** 1 School of Pharmacy, Changchun University of Chinese Medicine, Changchun 130117, China; jinye@ccucm.edu.cn (Y.J.); 23203070205@stu.ccucm.edu.cn (X.J.); 15804440604@163.com (D.F.); 15943431618@163.com (X.Z.); liuda@ccucm.edu.cn (D.L.); 2 Northeast Asian Institute of Traditional Chinese Medicine, Changchun University of Chinese Medicine, Changchun 130117, China; 3 Jilin Academy of Agricultural Sciences, Changchun 130033, China; jia07300909@163.com; 4 Public Experimental Center, Changchun University of Chinese Medicine, Changchun 130033, China

**Keywords:** *Ganoderma lucidum* glycoprotein, microemulsion, transdermal delivery, photoaging, antioxidant activity, skin repair

## Abstract

Background: Photoaging, induced by chronic ultraviolet (UV) exposure, is a multifactorial skin disorder characterized by oxidative stress, inflammation, and extracellular matrix degradation. *Ganoderma lucidum* glycoprotein (Gl-Gp) exhibits potent antioxidant activity, but its topical application is limited by poor transdermal permeability. This study aimed to develop a microemulsion-based system to enhance Gl-Gp delivery and evaluate its anti-photoaging efficacy. Methods: Gl-Gp was extracted and purified from G. lucidum fruiting bodies and structurally characterized for O-glycosidic linkages and O-GlcNAc modifications. Fourier-transform infrared (FT-IR) spectroscopy further confirmed the polysaccharide–protein complex structure of Gl-Gp. A water-in-oil Gl-Gp microemulsion was prepared and assessed in vitro for antioxidant and cytoprotective effects in HaCaT cells, including reactive oxygen species (ROS) reduction, mitochondrial membrane potential stabilization, and apoptosis inhibition. Transdermal penetration was compared with aqueous Gl-Gp. In vivo efficacy was evaluated in a UV-induced rat model by measuring skin morphology, histology, oxidative stress markers, matrix metalloproteinases, and proinflammatory cytokines. Results: The microemulsion enhanced Gl-Gp stability and transdermal delivery. In vitro, it reduced ROS, preserved mitochondrial function, and decreased apoptosis in HaCaT cells. In rats, topical application attenuated erythema and epidermal hyperplasia, promoted dermal restoration, increased SOD and GSH-Px activities, and decreased MDA, hydroxyproline, MMPs, and inflammatory mediators. Conclusions: The Gl-Gp microemulsion exerts antioxidant, anti-inflammatory, and anti-collagen-degrading effects, representing a promising strategy for transdermal delivery and topical prevention of photoaging.

## 1. Introduction

With the improvement of living standards, public interest in skin anti-aging and repair has been increasing. Skin aging is a multifaceted biological phenomenon, generally divided into two categories: intrinsic and extrinsic aging [1]. Intrinsic aging arises from unavoidable endogenous processes, such as the body’s natural senescence, while extrinsic aging is predominantly driven by external influences, including ultraviolet (UV) radiation, chemical pollutants, and tobacco use [2]. Among the various external stressors, skin aging induced by ultraviolet (UV) radiation is commonly termed photoaging. Frequent UV exposure can lead to a range of skin conditions, such as erythema, wrinkles, hyperpigmentation, and even skin cancer [3]. This effect is mainly attributed to the overproduction of reactive oxygen species (ROS), which disturbs the equilibrium between oxidative processes and the body’s antioxidant defenses [4].

Accumulation of ROS consumes both non-enzymatic antioxidants, such as ascorbic acid, tocopherols, panthenol, and glutathione, and enzymatic antioxidants, including glutathione peroxidase, catalase, and superoxide dismutase [5]. This disruption in redox homeostasis can trigger the MAPK signaling cascade, leading to elevated expression of matrix metalloproteinases (MMPs) that subsequently degrade collagen fibers. Moreover, elevated ROS levels result in abnormal activation of the Nrf2 and NF-κB pathways, ultimately impairing mitochondrial membrane potential and causing DNA damage and apoptosis [6].

*Ganoderma lucidum*, a medicinal mushroom with a long history of use, has been traditionally employed in China for millennia [7]. According to Shennong’s Classic of the Materia Medica, *G*. *lucidum* is neutral in nature and sweet in flavor. It is believed to tonify the blood, strengthen the spleen and stomach, calm the mind, enhance vitality, and delay aging. Traditionally, it has been employed to treat insomnia, fatigue, pulmonary deficiency-related coughing and wheezing, and palpitations. Pharmacologically, *G*. *lucidum*, belonging to the *Polyporaceae* family, demonstrates a wide range of biological activities, including protective effects on the heart, liver, and kidneys, as well as antitumor, immunomodulatory, and anti-aging properties [8].

Recent studies indicate that *G*. *lucidum* polysaccharides enhance the viability of UV-exposed human fibroblasts, attenuate cellular senescence, increase CICP protein levels, and inhibit MMP-1 expression [8]. Moreover, these polysaccharides reduce intracellular ROS levels, thereby protecting fibroblasts from oxidative damage. However, to date, no studies have reported the anti-photoaging effects of *G*. *lucidum* glycoproteins [9]. In addition, recent evidence suggests that glycoproteins derived from natural sources exhibit beneficial effects in skin-related therapies. For example, glycoproteins extracted from sea cucumber showed significant whitening and anti-wrinkle effects by inhibiting tyrosinase and elastase activities [10]. Dendrobium officinale glycoprotein was reported to promote fibroblast proliferation and accelerate wound healing by regulating extracellular matrix secretion via the EGFR/AKT/JAK-STAT pathways [11]. Moreover, sesame glycoproteins exerted photoprotective effects against UV-induced damage by suppressing MMP-1 expression and modulating MAPK/AKT signaling pathways [12]. These findings collectively suggest that natural glycoproteins possess promising therapeutic potential for skin repair and anti-aging applications, providing valuable insights into exploring the dermatological activity of *G*. *lucidum* glycoproteins.

Microemulsions are thermodynamically stable systems characterized by high industrial scalability, large interfacial areas, and excellent skin permeability and moisturizing properties. They are capable of efficiently traversing the dense architecture of the stratum corneum, thereby improving transdermal drug delivery. In this study, *G*. *lucidum* glycoprotein (Gl-Gp) was extracted and structurally characterized, and its protective effects against UV-induced skin photoaging were evaluated in a rat model. Given that Gl-Gp is a hydrophilic macromolecule with limited skin permeability, it was formulated into a water-in-oil (W/O) microemulsion to enhance its transdermal delivery efficiency [13]. Transdermal administration can improve therapeutic efficacy and bioavailability while minimizing adverse effects associated with oral delivery [14]. However, the major challenge for transdermal delivery lies in overcoming the skin’s barrier function. Thus, the development of efficient delivery systems capable of crossing this barrier is crucial.

Microemulsions improve transdermal drug uptake owing to their nanoscale particle size, distinct structural features, and beneficial interactions with the skin barrier. Wang et al. [15] and Tabosa et al. [16] demonstrated that microemulsions enhance skin permeation and increase epidermal and dermal drug accumulation, highlighting their transdermal delivery advantages. Moreover, Bhatia et al. [17] performed a 24-h in vitro skin permeation study using Franz diffusion cells with pig ear skin and observed that adapalene microemulsions substantially enhanced drug penetration in the stratum corneum (from 1.40 µg to 3.37 µg) and hair follicles (from 0.017 µg to 0.292 µg), providing a promising foundation for future applications.

**Figure 1:**
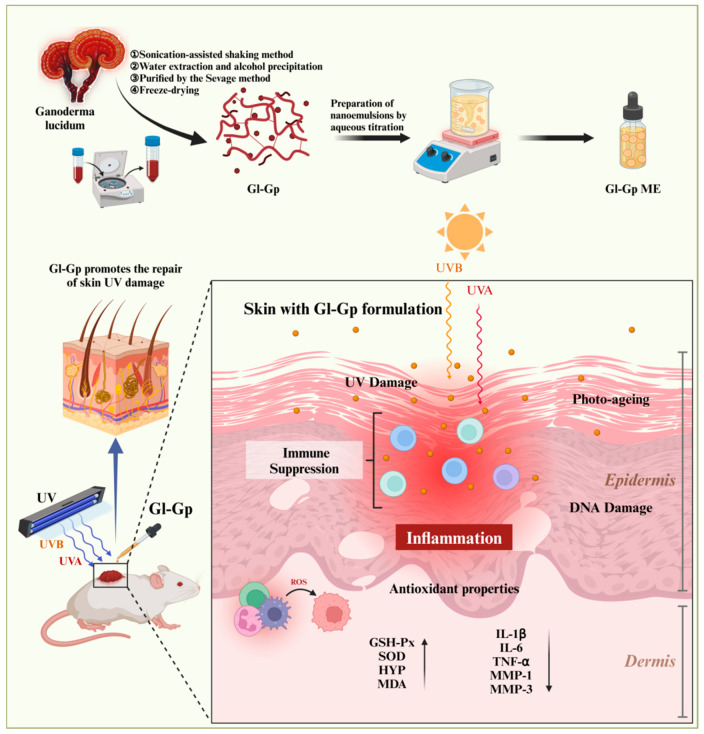
Schematic illustration of the preparation process and therapeutic mechanism of the *Ganoderma lucidum* glycoprotein (Gl-Gp) microemulsion-based transdermal delivery system for the treatment of UV-induced skin photoaging. Upward arrows indicate upregulation with improvement and antioxidant effects, while downward arrows represent downregulation of the measured factors.

Recent investigations have expanded the understanding of *Ganoderma lucidum* in skin anti-aging research. Lee et al. demonstrated that glycopeptide- and glycoprotein-enriched fractions derived from a multi-herbal formula containing *G*. *lucidum* exhibited anti-wrinkle and skin-firming effects by regulating extracellular matrix-related genes such as MMP-1, COL1A1, and HAS2 [18]. Similarly, Wang et al. reported that *G*. *lucidum* extract alleviated UVA-induced photoaging by maintaining mitochondrial homeostasis, reducing mtROS accumulation, and improving ATP synthesis [19]. These findings provide valuable evidence for the skin-protective effects of *G*. *lucidum* and its related bioactive components. Nevertheless, most existing studies have mainly emphasized biological activity, while aspects related to formulation stability, skin penetration, and transdermal delivery efficiency remain less explored. In light of these observations, the present study attempts to further explore this area by developing a Ganoderma glycoprotein-based microemulsion (Gl-Gp ME) designed to improve the stability, bioavailability, and skin permeability of glycoproteins. This formulation-oriented approach aims to complement previous biological studies and to provide additional experimental evidence on how delivery systems may enhance the topical anti-photoaging potential of *G*. *lucidum* glycoproteins. As shown in Figure 1, the schematic overview illustrates the preparation of the Ganoderma lucidum glycoprotein microemulsion (Gl-Gp ME) and its protective mechanism against UV-induced skin photoaging in rats, highlighting its role in improving transdermal delivery and promoting skin repair.

## 2. Results

### 2.1. Structure Characterization of Gl-Gp

To comprehensively characterize the molecular features of *Ganoderma lucidum* glycoprotein (Gl-Gp), a series of multidimensional analytical techniques was employed to elucidate its chemical composition and structural properties. Unlike small-molecule natural products that can be identified by GC–MS or ^1^H/^13^C NMR, Gl-Gp is a complex polysaccharide–protein conjugate, for which such techniques are not fully applicable. Therefore, alternative biochemical and spectroscopic approaches were performed to ensure accurate characterization.

**Figure 2:**
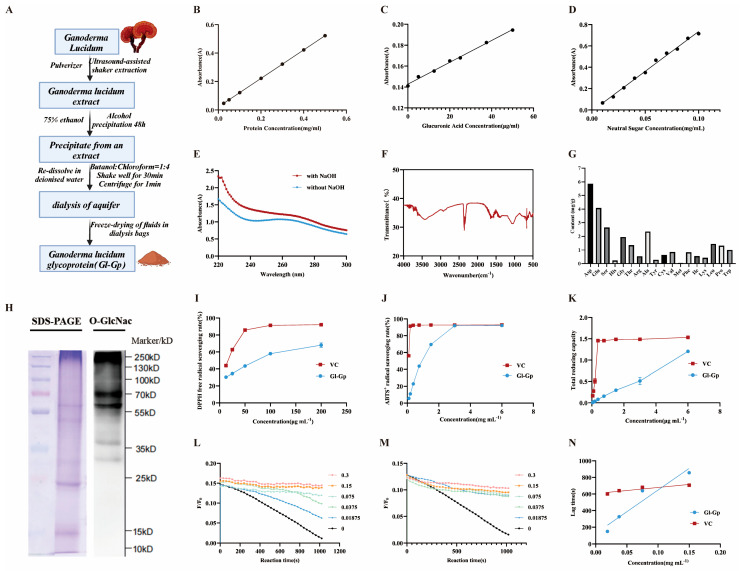
Structural characterization and in vitro antioxidant activities of *Ganoderma lucidum* glycoprotein (Gl-Gp). (**A**) Schematic representation of Gl-Gp extraction and purification. (**B**–**D**) Standard curves for quantifying (**B**) protein, (**C**) neutral sugars, and (**D**) acidic sugars. (**E**) β-Elimination assay assessing O-linked glycosylation. (**F**) FT-IR spectrum of Gl-Gp. (**G**) Amino acid composition analysis. (**H**) Identification of O-GlcNAc modification sites. (**I**–**K**) Antioxidant capacity evaluation: (**I**) DPPH radical scavenging, (**J**) ABTS^+^ radical scavenging, (**K**) total reducing power. (**L**,**M**) Kinetics of pyranine fluorescence bleaching for varying concentrations of (**L**) Gl-Gp and (**M**) vitamin C (VC). (**N**) Correlation between concentration and lag time for VC and Gl-Gp. Statistical significance was determined using one-way ANOVA with Tukey’s post hoc test. Data are presented as mean ± SD (n = 3).

Based on standard calibration curves for protein, glucose, and glucuronic acid (y = 0.001x + 0.1428, R^2^ = 0.9952) (Figure 2A–D), quantitative analysis revealed that Gl-Gp contains 45.56 ± 0.79% protein, 10.18 ± 0.29% neutral sugars, and 22.69 ± 0.22% acidic sugars, indicating that Gl-Gp is a typical glycoprotein composed of tightly associated polysaccharide chains and protein cores. SDS-PAGE analysis further confirmed the purity and molecular weight distribution of Gl-Gp, supporting its identity as a defined glycoprotein fraction rather than a crude extract.

To determine the nature of the glycosidic linkages, Gl-Gp was subjected to a β-elimination assay under alkaline conditions. The NaOH-treated group exhibited a marked increase in UV absorption at 240 nm (Figure 2E), suggesting that the sugar moieties are O-glycosidically linked to the protein backbone via threonine and/or serine residues—linkages critical for glycoprotein stability and bioactivity. Further confirmation of post-translational glycosylation was achieved by Western blotting with an anti-O-GlcNAc antibody (Figure 2H). A distinct O-GlcNAc signal was detected within the 15–25 kDa molecular weight range, confirming the presence of O-GlcNAc modifications on serine (Ser) and threonine (Thr) residues. These modifications are known to influence protein folding, intracellular trafficking, and biological functions.

Fourier-transform infrared (FT-IR) spectroscopy (Figure 2F) provided additional confirmation of the polysaccharide–protein complex structure of Gl-Gp. A broad peak near 3400 cm^−1^ was assigned to O–H and N–H stretching vibrations, whereas the signals at 1640 cm^−1^ and 1540 cm^−1^ corresponded to the amide I and amide II bands of the protein, respectively. Additionally, the absorption at 1062 cm^−1^ was assigned to C–O–C stretching vibrations within the pyranose rings of polysaccharides, collectively confirming the structural integration of sugar and protein components.

Amino acid composition analysis (Figure 2G) showed that Gl-Gp comprises 18 amino acids, including 9 essential amino acids (e.g., leucine, isoleucine, and valine), with particularly high levels of aspartic acid (Asp, 5.86 mg/g) and glutamic acid (Glu, 4.08 mg/g), both of which may contribute to structural stability through electrostatic interactions. Moreover, the abundant presence of serine (Ser, 2.65 mg/g) and threonine (Thr, 1.36 mg/g) correlates well with the identified O-glycosylation sites.

In summary, the combined results from FT-IR, SDS-PAGE, amino acid composition analysis, β-elimination assay, and O-GlcNAc immunoblotting provide a comprehensive structural validation of Gl-Gp. These molecular features—O-glycosidic linkages, O-GlcNAc modifications, pyranose-based polysaccharide structures, and a diverse amino acid profile—establish a solid biochemical foundation for its antioxidant and anti-photoaging activities.

### 2.2. In Vitro Antioxidant Activity of Gl-Gp

The DPPH radical scavenging activity of Gl-Gp was assessed, and the findings (Figure 2I) revealed a marked, dose-dependent enhancement in scavenging capacity over the concentration range of 12.5–200 μg/mL. The IC_50_ values for vitamin C (VC) and Gl-Gp were 14.72 μg/mL and 62.80 μg/mL, respectively, indicating that Gl-Gp possesses strong DPPH radical scavenging activity, albeit slightly lower than that of VC. Similarly, Gl-Gp exhibited robust ABTS^+^ radical scavenging activity (Figure 2J), with a clear positive correlation between concentration and activity over the range of 0.09–6 mg/mL. At 3 mg/mL, the scavenging rate of Gl-Gp reached 92.05%, comparable to that of VC at the same concentration. The IC_50_ values for VC and Gl-Gp in this assay were 0.019 mg/mL and 0.804 mg/mL, respectively, further confirming Gl-Gp’s potent antioxidant potential. The total reducing power of Gl-Gp was assessed via the ferric ion (Fe^3+^) reduction method (Figure 2K). Under acidic conditions, K_3_Fe(CN)_6_ reacts with Fe^3+^ to generate a colored complex, and the resulting absorbance correlates with reducing capacity. Although Gl-Gp showed a lower reducing power than VC, a clear dose-dependent increase was observed over the 0.09–6 mg/mL range, suggesting Gl-Gp’s ability to act as an effective electron donor. In addition, the anti-lipid peroxidation capacity of Gl-Gp was evaluated (Figure 2L–N). The suppression of lipid peroxidation showed a positive correlation with the slope of the concentration–lag time curve, indicating the ability of Gl-Gp to scavenge AAPH-generated peroxyl radicals. At excitation and emission wavelengths of 456 nm and 512 nm, respectively, the regression equation for vitamin C (VC) was Y = 743.08X + 605.5 (slope: 743.08), whereas that for Gl-Gp was Y = 5228.5X + 125.38 (slope: 5228.5). The substantially greater slope observed for Gl-Gp indicates superior inhibitory activity against lipid peroxidation compared to VC. Exposure to ultraviolet (UV) radiation triggers excessive generation of reactive oxygen species (ROS), such as superoxide anions, singlet oxygen, hydroxyl radicals, and hydrogen peroxide. These ROS drive oxidative stress and impair both the structural and functional integrity of skin tissues. This work systematically assessed the antioxidant potential of Gl-Gp through a combination of DPPH and ABTS^+^ radical neutralization assays, measurement of reducing power, and evaluation of lipid peroxidation suppression. The results provide strong theoretical support for the use of Gl-Gp in photoaging prevention and repair. In conclusion, Gl-Gp exhibits excellent antioxidant activity, particularly in DPPH and ABTS^+^ radical scavenging and inhibition of lipid peroxidation, with clear dose–effect relationships. These findings suggest its strong potential for applications in antioxidant therapy and photoaging intervention.

### 2.3. Gl-Gp Demonstrates Exceptional Biocompatibility

**Figure 3:**
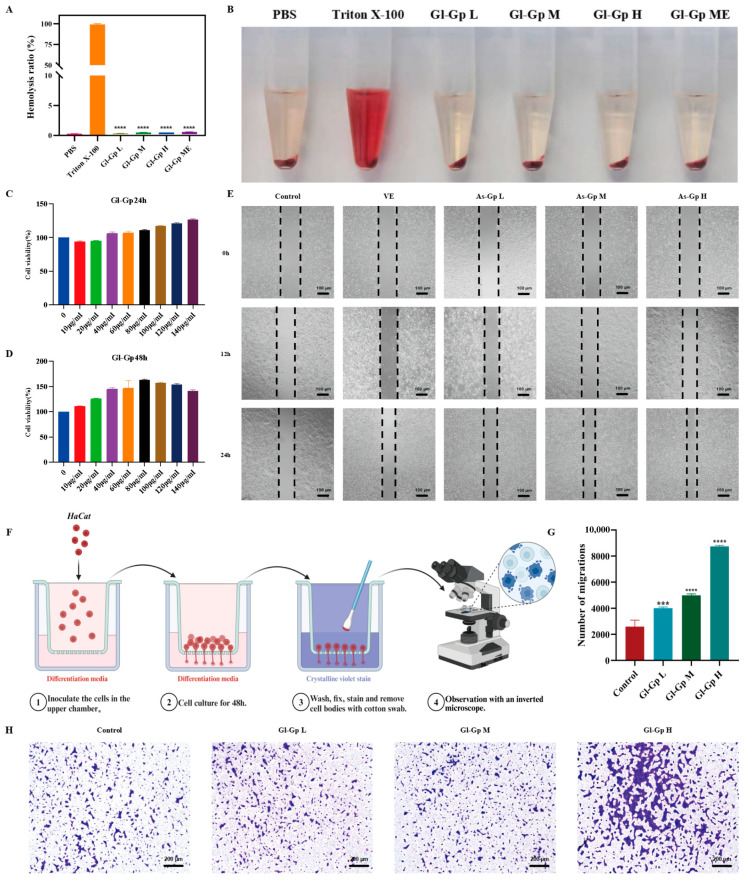
Biocompatibility and pro-migratory effects of *Ganoderma lucidum* glycoprotein (Gl-Gp) on HaCaT cells (Gl-Gp) on HaCaT cells. In vitro hemolysis was assessed (**A**,**B**), and CCK-8 assays were performed to determine cell proliferation rates (**C**,**D**). Representative images from the scratch assay at 12 h and 24 h post-wounding are shown in (**E**), while (**F**–**H**) display the results of the Transwell migration assay at different Gl-Gp concentrations. Data are presented as mean ± SD (*n* = 3). Statistical significance was analyzed using one-way ANOVA with Tukey’s post hoc test. *** *p* < 0.001, **** *p* < 0.0001 vs. model group.

The CCK-8 assay, a widely used and reliable method for assessing cell proliferation and viability, was employed to evaluate the cytocompatibility of Gl-Gp [20]. As shown in Figure 3C,D, after 24 h of incubation, the viability of HaCaT cells in all treatment groups exceeded 90%, indicating minimal cytotoxicity. At 48 h, Gl-Gp at concentrations of 40, 80, and 120 μg/mL significantly promoted cell proliferation, with relative viability rates of 146.47%, 157.07%, and 150.01%, respectively. These findings suggest that Gl-Gp not only lacks cytotoxic effects but also enhances cellular proliferation.

To evaluate the hemocompatibility of Gl-Gp, a hemolysis assay was conducted, employing Triton X-100 as a positive control and PBS as a negative control. As shown in Figure 3A,B, red blood cells in the positive control group experienced extensive hemolysis, resulting in a distinctly red supernatant. In contrast, Gl-Gp caused only a minor, concentration-dependent rise in hemolysis, with rates remaining below 5% across all tested concentrations, confirming its superior hemocompatibility.

Collectively, the above findings demonstrate that Gl-Gp possesses outstanding biocompatibility, with both high cytocompatibility and hemocompatibility, supporting its potential for safe biomedical applications.

### 2.4. Gl-Gp Promotes Migration of HaCaT Cells

The effect of Gl-Gp on HaCaT cell migration was assessed using a scratch wound assay. After 12 h of treatment, cells exposed to Gl-Gp showed a pronounced reduction in wound area compared to the untreated control (Figure 3E). By 24 h, scratch gaps in Gl-Gp-treated cultures exhibited substantial closure. Specifically, the cell migration rates at Gl-Gp concentrations of 40, 80, and 120 μg/mL were 67.92%, 77.07%, and 80.83%, respectively, whereas the control group displayed a migration rate of 50.28%. These results indicate that Gl-Gp enhances HaCaT cell migration in a concentration-dependent manner. To further support these observations, a Transwell migration assay was conducted, confirming that Gl-Gp also promoted three-dimensional (3D) migration of HaCaT cells (Figure 3F–H). Quantitative evaluation of the 3D migration data was consistent with the results of the 2D scratch assay. In summary, the in vitro results indicate that Gl-Gp significantly enhances HaCaT keratinocyte migration in a concentration-dependent manner.

### 2.5. Gl-Gp Promotes RAW264.7 Cell Viability, Mitigates UVB-Induced (280–320 nm) Apoptosis and Mitochondrial Dysfunction in HaCaT Cells, and Reduces Intracellular ROS Accumulation Resulting from UVB Exposure

**Figure 4:**
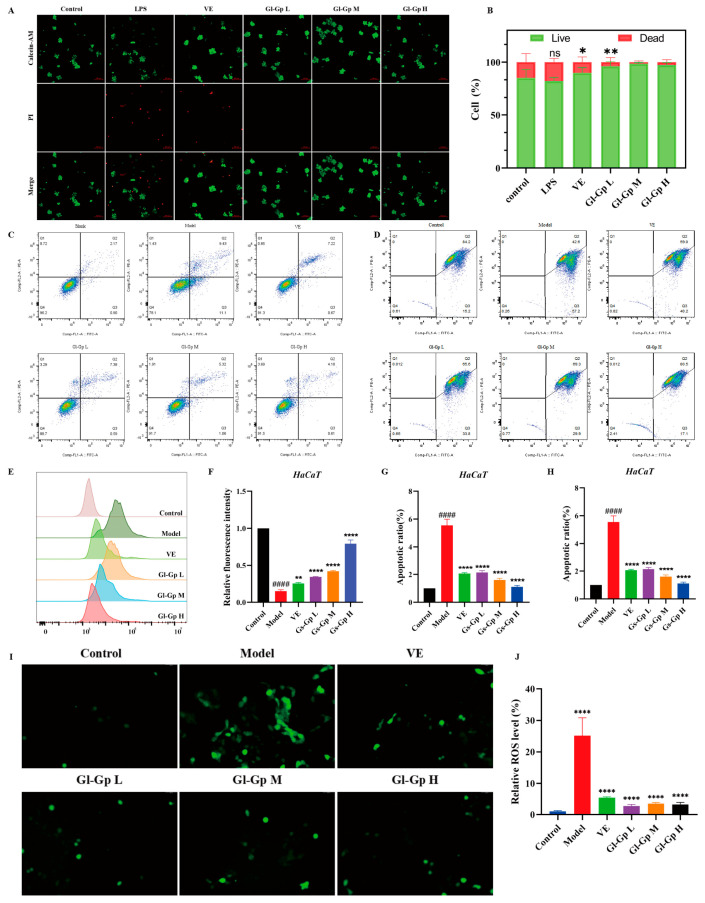
Protective effects of *Ganoderma lucidum* glycoprotein (Gl-Gp) on LPS-induced cytotoxicity in RAW264.7 cells and UVB-induced apoptosis, mitochondrial dysfunction, and ROS accumulation in HaCaT cells. Live/dead staining of RAW264.7 cells after LPS treatment is shown in (**A**,**B**). Apoptosis of HaCaT cells was analyzed using flow cytometry (**C**–**F**), and mitochondrial membrane potential was evaluated via JC-1 staining combined with flow cytometry (**D**–**G**). ROS levels in HaCaT cells following UVB irradiation were assessed by fluorescence staining (**E**–**H**), and intracellular ROS was further visualized using immunofluorescence imaging (**I**,**J**). Data are presented as mean ± SD (*n* = 3). Statistical significance was analyzed using one-way ANOVA with Tukey’s post hoc test. * *p* < 0.05, ** *p* < 0.01, **** *p* < 0.0001 vs. model group. #### *p* < 0.0001 vs. Control group.

Calcein-AM/PI double staining was used to evaluate the viability of RAW264.7 cells exposed to lipopolysaccharide (LPS) in all experimental groups. As illustrated in Figure 4A,B, cells treated with LPS showed a marked increase in red fluorescence, indicative of substantial cell death, accompanied by a notable decrease in green fluorescence relative to the control group. In contrast, treatment with Gl-Gp substantially decreased red fluorescence, while the number of viable cells nearly matched that of the control group, indicating a protective effect against LPS-induced cytotoxicity. Moreover, Gl-Gp treatment enhanced cell viability in a concentration-dependent manner, emphasizing its capacity to alleviate LPS-induced cellular damage. Apoptosis in HaCaT cells induced by UVB exposure was evaluated using Annexin V-FITC/PI dual staining combined with flow cytometry [21]. The control group (without UVB exposure) exhibited an apoptosis rate of 3.07%, whereas UVB irradiation significantly elevated the apoptotic cell percentage to 20.53% (Figure 4C,F). Treatment with Gl-Gp at 40, 80, and 120 μg/mL decreased apoptosis rates to 7.97%, 6.38%, and 4.79%, respectively. Notably, the medium- and high-dose Gl-Gp groups demonstrated greater anti-apoptotic effects than the positive control (vitamin E) and significantly lower apoptosis rates compared to the UVB group, indicating effective protection against UVB-induced apoptosis. To investigate mitochondrial damage induced by UVB and the protective effects of Gl-Gp, JC-1 staining was employed to evaluate mitochondrial membrane potential (Δψm) and examine UVB-induced mitochondrial damage as well as the protective effects of Gl-Gp (Figure 4D,G). In the control group, cells exhibited intense red fluorescence, reflecting JC-1 aggregation and intact mitochondrial membrane potential (Δψm). In contrast, UVB-irradiated cells displayed reduced red fluorescence and elevated green fluorescence (JC-1 monomers), indicating mitochondrial depolarization. Treatment with Gl-Gp restored red fluorescence intensity in a concentration-dependent manner. Notably, cells treated with a high dose of Gl-Gp (120 μg/mL) showed a 2.3-fold increase (*p* < 0.01) in the red/green fluorescence ratio compared to the UVB group, indicating effective stabilization of mitochondrial membrane potential. The impact of Gl-Gp on intracellular reactive oxygen species (ROS) induced by UVB irradiation was further assessed using DCFH-DA staining (Figure 4E,H–J). Control cells showed minimal green fluorescence, whereas UVB exposure caused a marked increase in fluorescence intensity, indicating elevated ROS production. Gl-Gp treatment reduced green fluorescence intensity in a dose-dependent manner, confirming its ability to suppress UVB-induced ROS accumulation and mitigate oxidative stress. Collectively, Gl-Gp confers dual protective effects by directly scavenging excessive ROS and preserving mitochondrial membrane potential, thereby mitigating oxidative damage. These findings provide mechanistic insights into the anti-photoaging properties of Gl-Gp.

This study demonstrates that Gl-Gp effectively stabilizes the mitochondrial membrane potential (ΔΨm) and reduces mitochondrial ROS levels in UVB-induced HaCaT cells. These results are consistent with the findings of Wang et al., who reported that *Ganoderma lucidum* extract (GLE) preserves mitochondrial function in photoaging models [19]. Building upon this foundation, the present study further shows that incorporation of Gl-Gp into a microemulsion delivery system allows the active component to maintain its physiological activity even after transdermal administration. These findings provide a new perspective for understanding the mitochondrial protective mechanisms of *G*. *lucidum* and suggest potential technological strategies for enhancing its practical applications in skin protection.

### 2.6. Preparation and Characterization of Gl-Gp ME

**Figure 5:**
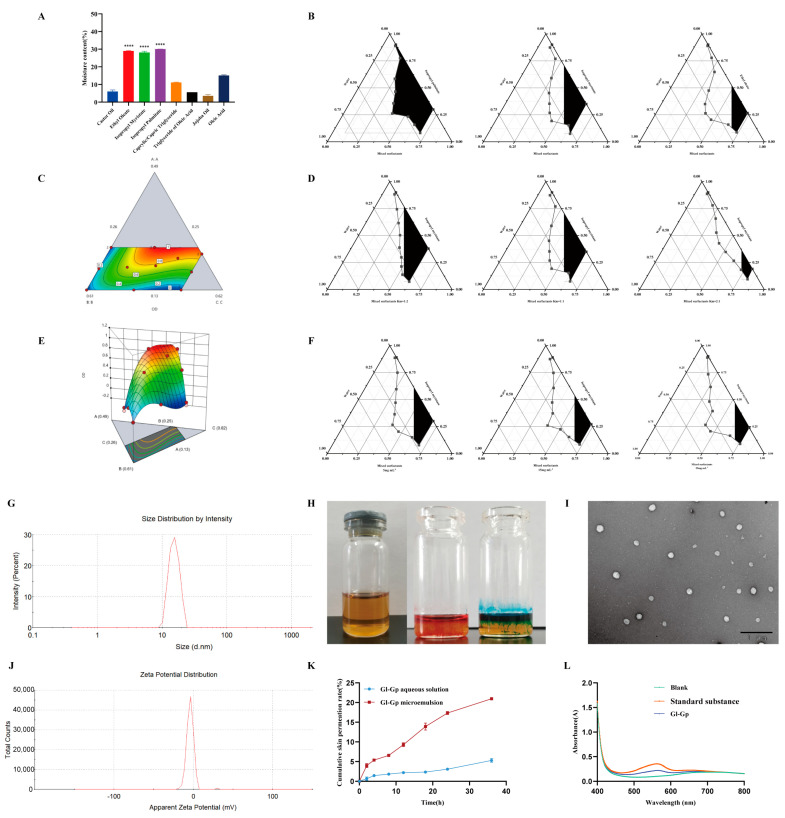
Preparation, characterization, and enhanced transdermal delivery of *Ganoderma lucidum* glycoprotein (Gl-Gp) water-in-oil (W/O) microemulsion. Water retention capacities of different oil phases are presented in (**A**). Optimization of the Gl-Gp microemulsion was performed using pseudo-ternary phase diagrams, single-factor experiments, and a D-optimal mixture design (**B**–**F**). The microemulsion particle size distribution is shown in (**G**), and its appearance is displayed in (**H**). Transmission electron microscopy (TEM) images of microemulsion droplets are shown in (**I**), and zeta potential measurements are presented in (**J**). The in vitro skin permeation profile and UV absorption spectrum of the microemulsion are shown in (**K**,**L**). Data are presented as mean ± SD (*n* = 3). Statistical significance was assessed using one-way ANOVA followed by Tukey’s post hoc test. **** *p* < 0.0001 vs. model group.

*Ganoderma lucidum* glycoprotein (Gl-Gp) is a water-soluble macromolecule with promising anti-photoaging properties; however, its transdermal absorption is limited by its hydrophilic nature. A water-in-oil (W/O) microemulsion was prepared using the water titration method to improve Gl-Gp’s percutaneous permeability and stability. Initially, different oil phases were assessed for their water-holding capacity, with isopropyl myristate identified as the optimal choice (Figure 5A). The formulation was subsequently optimized through pseudo-ternary phase diagrams, single-factor experiments, and D-optimal mixture design (Figure 5B–F). The final W/O microemulsion formulation contained 36.5% isopropyl myristate as the oil phase, 37.5% surfactant mixture of soybean phospholipids and ethanol at a Km ratio of 1:1, and 26.0% aqueous phase, with Gl-Gp at 15 mg/mL. The prepared microemulsion demonstrated high encapsulation efficiency (96.17 ± 0.11%) and drug loading capacity (3.70 ± 0.07 mg/g). It was visually clear and transparent, with a mean droplet size of approximately 19 ± 2.76 nm (Figure 5G,H), and confirmed as a W/O-type system via dye solubility and dilution tests. Transmission electron microscopy (TEM) images (Figure 5I,J) revealed spherical, uniformly distributed, and well-dispersed droplets. The formulation’s pH was within the physiological range compatible with skin, indicating good biocompatibility. Furthermore, the microemulsion demonstrated excellent physical stability, showing no phase separation or precipitation over 30 days, with encapsulation efficiency and drug loading remaining stable throughout the period. In vitro skin permeation studies showed that the cumulative transdermal permeation of the Gl-Gp microemulsion reached 20.98 ± 0.34% over 36 h, significantly higher than that of the aqueous Gl-Gp solution (5.31 ± 0.49%) (Figure 5K,L), confirming the enhanced permeation capability of the microemulsion. In summary, the developed Gl-Gp-loaded W/O microemulsion exhibits excellent stability, biocompatibility, and markedly improved transdermal delivery, providing a strong foundation for its application in transdermal drug delivery systems.

### 2.7. Protective Effect of Gl-Gp on Skin Damage Caused by UVA (320–400 nm) + UVB

**Figure 6:**
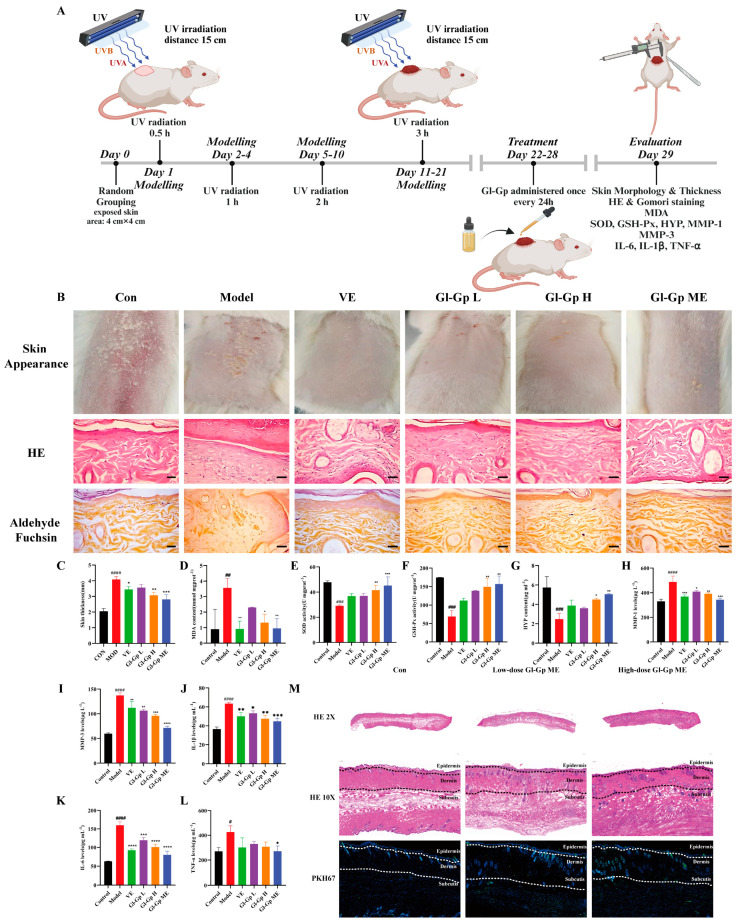
Protective effects of *Ganoderma lucidum* glycoprotein microemulsion (Gl-Gp ME) on UVA + UVB-induced skin damage in rats. (**A**) Experimental timeline for establishing the UVA + UVB-induced skin photodamage model. (**B**) Representative dorsal skin images of each treatment group after 21 days of UVA + UVB exposure, including hematoxylin and eosin (H&E) and aldehyde fuchsin staining. Scale bar: 100 µm. (**C**) Quantitative analysis of total skin thickness. (**D**–**L**) Measurement of oxidative stress markers and inflammatory mediators in rat skin, including malondialdehyde (MDA), superoxide dismutase (SOD), glutathione peroxidase (GSH-Px), hydroxyproline (HYP), matrix metalloproteinases (MMP-1, MMP-3), and proinflammatory cytokines (IL-1β, IL-6, TNF-α). (**M**) Representative H&E-stained images of epidermis, dermis, and subcutaneous tissue in the normal group, low-dose Gl-Gp ME group, and high-dose Gl-Gp ME group, along with immunofluorescence staining showing drug distribution in the skin. Data are expressed as mean ± SD (*n* = 3). Statistical significance was determined by one-way ANOVA followed by Tukey’s post hoc test. * *p* < 0.05, ** *p* < 0.01, *** *p* < 0.001, **** *p* < 0.0001 vs. model group. # *p* < 0.05, ## *p* < 0.01, ### *p* < 0.001, #### *p* < 0.0001 vs. Control group.

After UVA + UVB irradiation, rats received topical treatment with Gl-Gp for seven consecutive days (Figure 6A). Representative dorsal skin images were captured on day 7 (Figure 6B). Rats exposed to UVA + UVB exhibited classic photodamage features such as erythema, edema, scabbing, and epidermal rupture, whereas the Gl-Gp-treated groups and the positive control (vitamin E, VE) displayed varying levels of improvement. Notably, the high-dose Gl-Gp and Gl-Gp microemulsion (ME) groups demonstrated superior recovery, with minimal erythema and scabbing observed. UVA + UVB exposure caused significant skin dehydration, evidenced by decreased skin water content. Histological evaluation of skin structure was conducted using hematoxylin and eosin (H&E) and aldehyde fuchsin staining (Figure 6B). The control group displayed a thin epidermis with intact dermal-epidermal junctions. In contrast, UVA + UVB exposure induced epidermal thickening and disrupted dermal architecture. Treatment with Gl-Gp and Gl-Gp ME ameliorated these pathological changes, particularly in the high-dose and ME groups, where collagen and elastic fibers were well-organized and resembled those of the control group. Quantitative analysis confirmed significant reductions in epidermal and total skin thickness following treatment (Figure 6C), indicating anti-inflammatory effects. Both VE and Gl-Gp ME groups showed similar improvements in skin hydration and thickness compared to the UVA + UVB group. Oxidative stress markers were also assessed. UVA + UVB exposure markedly elevated malondialdehyde (MDA) levels while reducing the activities of superoxide dismutase (SOD) and glutathione peroxidase (GSH-Px). Treatment with Gl-Gp and Gl-Gp ME reversed these changes in a dose-dependent manner (*p* < 0.01), with antioxidant enzyme activities in the high-dose group approaching control levels. Furthermore, hydroxyproline (HYP) content, which was markedly reduced by UVA + UVB exposure, was significantly restored in the Gl-Gp-treated groups, suggesting enhanced collagen synthesis (Figure 6D–G). ELISA analysis revealed that UVA + UVB exposure increased the levels of matrix metalloproteinases (MMP-1 and MMP-3) and proinflammatory cytokines (IL-1β, IL-6, TNF-α). Both Gl-Gp and Gl-Gp microemulsion (ME) significantly inhibited these inflammatory mediators, with the strongest effects seen in the high-dose group, where TNF-α levels approached baseline (Figure 6H–L). In summary, Gl-Gp ME effectively mitigated UVA + UVB-induced skin damage by reducing oxidative stress, inhibiting inflammation, and modulating MMP expression. It restored epidermal integrity and promoted remodeling of collagen and elastic fibers. These therapeutic effects were comparable to those of commercially available VE, demonstrating that Gl-Gp, even as a single-component agent, holds strong potential for treating UVA + UVB-induced photodamage. To evaluate the transdermal distribution of Gl-Gp microemulsion (Gl-Gp ME), immunofluorescence staining was performed using PKH67-labeled Gl-Gp ME. Six hours after topical application, rats were euthanized, and dorsal skin samples were collected, cryosectioned, and observed under a fluorescence microscope (Figure 6M). In the low-dose Gl-Gp ME group, green fluorescence was detectable in both the epidermis and dermis, indicating that a small amount of Gl-Gp had already penetrated through the stratum corneum into the dermal layer by the sixth hour. In contrast, the high-dose Gl-Gp ME group showed markedly stronger green fluorescence in the dermis at the same time point, demonstrating enhanced drug penetration. These observations suggest that the Gl-Gp ME formulation not only improves the permeability of Gl-Gp through the skin barrier but also facilitates its delivery into deeper dermal layers. Moreover, the fluorescence distribution pattern indicated a dose-dependent effect on dermal accumulation, with higher concentrations of Gl-Gp ME resulting in more extensive penetration and increased local bioavailability in the dermis. The partial retention of Gl-Gp in the epidermis further implies sustained release within the skin layers, which may contribute to prolonged therapeutic activity. Collectively, these results confirm that the W/O microemulsion system effectively enhances the percutaneous delivery of Gl-Gp and enables its distribution into both superficial and deeper skin layers, supporting its potential for topical anti-photoaging applications.

In vivo experiments revealed that Gl-Gp ME markedly increased the hydroxyproline (HYP) content in skin tissues and downregulated the expression of MMP-1 and MMP-3. These results are in agreement with the findings of Lee et al., who reported comparable collagen-promoting and MMP-1-suppressing effects using a multi-component glycoprotein-enriched fraction (GEF) containing *Ganoderma lucidum* [18]. Such consistency supports the notion that *G*. *lucidum*-derived glycoproteins possess promising potential for further development as bioactive agents in skin anti-aging applications.

## 3. Discussion

In this study, we developed a *Ganoderma lucidum* glycoprotein (Gl-Gp)-loaded microemulsion (Gl-Gp ME) and systematically evaluated its biocompatibility, wound healing potential, and protective effects against UV-induced skin photodamage. The results demonstrated that Gl-Gp exhibits excellent biosafety, as evidenced by negligible hemolytic activity and its ability to significantly promote HaCaT cell proliferation and migration. Gl-Gp demonstrates potential as a bioactive agent for skin regeneration and repair.

Moreover, the Gl-Gp ME formulation showed efficacy in vivo. In a UVA + UVB-induced skin photodamage rat model, Gl-Gp ME alleviated visible skin damage and contributed to the restoration of epidermal and dermal structures, as shown by histological staining. Quantitative analyses revealed that Gl-Gp ME reduced oxidative stress markers such as MDA while elevating antioxidant enzyme activities (SOD, GSH-Px). Additionally, it mitigated extracellular matrix degradation by downregulating MMP-1 and MMP-3 expression, and suppressed inflammatory cytokines (IL-1β, IL-6, TNF-α), highlighting its multi-targeted therapeutic potential in managing photodamage.

The observed protective effects are likely attributed to the synergistic interaction of multiple components within Gl-Gp, which may function through antioxidative, anti-inflammatory, and matrix-preserving mechanisms. Previous studies have reported that glycoproteins from *Ganoderma lucidum* exert protective effects via the modulation of MAPK and NF-κB pathways. Although the present study did not focus on delineating the molecular mechanisms underlying the observed effects, the combined in vitro and in vivo results indicate that the photoprotective activity of the Gl-Gp microemulsion is likely mediated through the regulation of oxidative stress-associated signaling pathways. Furthermore, the microemulsion delivery system appears to enhance formulation stability and local bioavailability, thereby contributing to the improved therapeutic outcomes observed in the photoaging model.

However, several considerations remain. The intrinsic physicochemical properties of Gl-Gp, including high molecular weight, strong hydrophilicity, and extensive glycosylation, pose challenges for deep skin penetration. While the W/O microemulsion may facilitate delivery to the viable epidermis, conventional formulations are unlikely to achieve substantial dermal penetration. To address this translational gap, we performed fluorescence-labeled frozen skin section experiments in rats, which provided preliminary evidence that Gl-Gp ME can reach viable epidermal cells and partially the dermis. Nevertheless, these findings are preliminary, and the mechanistic implications should be interpreted cautiously. Future studies should explore advanced or hybrid delivery strategies, such as microneedles, electroporation, ultrasound, or nanocarriers specifically engineered for macromolecular transport. Furthermore, to more accurately translate these findings, future investigations will employ more physiologically relevant models, including human skin organoids, ex vivo skin explants, or in vivo wound-healing/UV-damage models, to validate both efficacy and mechanistic pathways [22,23,24,25,26,27,28].

Importantly, while histological and biochemical analyses confirmed structural restoration of UV-damaged skin, this study did not assess functional skin properties such as elasticity or firmness, which are critical indicators of photoaging improvement. Future studies should incorporate non-invasive biophysical techniques (e.g., cutometry or suction-based elasticity tests) to quantify mechanical recovery and correlate it with histological and molecular outcomes, providing a more comprehensive evaluation of Gl-Gp ME’s reparative potential.

In summary, Gl-Gp ME exhibits antioxidant, anti-inflammatory, and anti-matrix degradation activities, supporting its potential to mitigate UV-induced skin injury. Future work should focus on cautious interpretation of mechanistic findings, elucidating molecular mechanisms in physiologically relevant models, optimizing formulation stability, and evaluating efficacy in advanced skin models or clinical settings.

## 4. Materials and Methods

### 4.1. Materials and Reagents

*Ganoderma lucidum* glycoprotein (Gl-Gp) was purchased from Shanzhiyuan Ecological Agriculture Co., Ltd. (Songyuan, China). The bicinchoninic acid (BCA) protein assay kit and Annexin V-FITC/propidium iodide (PI) apoptosis detection kit were obtained from Beyotime Biotechnology (Shanghai, China, Cat. No. C0062, C1062). D-(+)-Glucose anhydrous, phenol, sulfuric acid, D-glucuronic acid, m-hydroxydiphenyl, 1,1-diphenyl-2-picrylhydrazyl (DPPH), pyranine, ABTS^+^, and vitamin C were purchased from Yuanye Biotechnology (Shanghai, China, Batch No. provided by supplier). Sodium hydroxide was supplied by Beijing Chemical Works Co., Ltd. (Beijing, China), and Coomassie Brilliant Blue was purchased from Solarbio (Beijing, China). SDS-PAGE gel fast preparation kits were obtained from Melonepharma (Dalian, China). Absolute ethanol, chloroform, n-butanol, potassium ferricyanide, trichloroacetic acid, ferric chloride, and 2,2’-azobis(2-methylpropionamidine) dihydrochloride (AAPH) were provided by Xintong Fine Chemical Co., Ltd. (Tianjin, China). A 0.1 kDa dialysis bag was purchased from RWD Life Science (Shenzhen, China). Enhanced BCA protein assay, malondialdehyde (MDA), total superoxide dismutase (SOD), glutathione peroxidase (GSH-Px), and hydroxyproline (HYP) assay kits were obtained from Nanjing Jiancheng Bioengineering Institute (Nanjing, China). ELISA kits for TNF-α, IL-1β, IL-6, MMP-1, and MMP-3 were purchased from Jiangsu Meimian Industrial Co., Ltd. (Nanjing, China). Soybean phospholipid was supplied by Awaitu Pharmaceutical Technology (Shanghai, China). Murine macrophage RAW264.7 cells were obtained from Suzhou, China (Cell bank/source: specify if available), and human immortalized keratinocytes (HaCaT) were acquired from the BeNa Culture Collection (Suzhou, China, Cat. No. BNCC337265). HaCaT cells were maintained in Dulbecco’s Modified Eagle Medium (DMEM, HyClone, Logan, UT, USA, Version 4.0) supplemented with 10% fetal bovine serum (FBS, HyClone, Version 2.0) and 1% penicillin/streptomycin (Gibco, Grand Island, NY, USA, Version 3.0).

### 4.2. Animals and Cell Lines

Female Sprague-Dawley (SD) rats weighing 180–200 g were obtained from Changchunshi Yisi Experimental Animals Technology Co., Ltd. (Jilin, China; license no. SCXK[ji]-2020-0002). Rats were housed at the Changchun University of Chinese Medicine under controlled conditions (25 ± 1 °C, 12-h light/dark cycle) with ad libitum access to standard chow and water. All experimental procedures were approved by the Ethics Committee of Changchun University of Chinese Medicine and conducted in strict accordance with the National Research Council’s Guide for the Care and Use of Laboratory Animals.

Human keratinocyte HaCaT cells and rat HaCat fibroblasts were obtained from the China Center for Type Culture Collection (Wuhan, China). Cells were cultured in Dulbecco’s Modified Eagle Medium (DMEM) supplemented with 10% fetal bovine serum (FBS), 100 U/mL penicillin, and 100 μg/mL streptomycin, and incubated at 37 °C in a humidified atmosphere containing 95% air and 5% CO_2._

### 4.3. Preparation of Gl-Gp and Gl-Gp ME

The fruiting bodies of *Ganoderma lucidum* were initially ground and sieved through a 4-mesh screen to yield a consistent powder. The effective components were then extracted using an ultrasound-assisted shaking method. The extract was treated with ethanol to precipitate free sugars, followed by Sevage treatment (n-butanol–chloroform = 1:4) to remove residual proteins. The resulting aqueous fraction was collected, dialyzed to eliminate small-molecular-weight impurities, and lyophilized to obtain purified *Ganoderma lucidum* glycoprotein (Gl-Gp).

A 15 mg/mL Gl-Gp solution was incorporated as the aqueous phase, with IPM as the oil phase and a soybean phospholipid–ethanol mixture as the surfactant system (Km = 1). The components were combined at a mass ratio of 0.260:0.365:0.375, and the aqueous phase was added dropwise to produce a uniform microemulsion. The prepared Gl-Gp microemulsion was transparent and stable, with an encapsulation efficiency of 96.17 ± 0.11% and a drug loading of 3.70 ± 0.07 mg/g, demonstrating high encapsulation and loading capacity.

### 4.4. Characterization of Gl-Gp and Gl-Gp ME

Gl-Gp protein content was measured by the BCA assay at 562 nm, neutral sugars by the phenol–sulfuric acid method at 490 nm, and uronic acids via the m-hydroxydiphenyl assay, enabling comprehensive compositional characterization. The type of glycosidic linkage between sugar and peptide was evaluated by β-elimination. Specifically, Gl-Gp was dissolved separately in 0.4 mol/L NaOH and distilled water, incubated at 60 °C for 30 min, and scanned in the UV range of 220–300 nm. Changes in absorbance at 240 nm were used to assess the presence of O-glycosidic linkages.

O-GlcNAc modification sites of Gl-Gp were analyzed by Western blotting, with proteins denatured at 95 °C for 10 min and separated via SDS-PAGE, facilitating post-translational modification characterization. Proteins were transferred to NC membranes and blocked with 5% non-fat milk in TBST for 1 h at room temperature to prevent nonspecific binding. The membrane was washed thrice with TBST and incubated overnight at 4 °C with primary anti-O-GlcNAc antibody in 2.5% BSA/TBST to detect O-GlcNAc modifications. The membrane was incubated with HRP-conjugated secondary antibody for 1 h at room temperature, followed by chemiluminescent substrate treatment and signal detection using an imaging system. This method allows sensitive detection of O-GlcNAc-modified sites and facilitates the structural characterization of Gl-Gp.

FTIR analysis of Gl-Gp was performed using the KBr pellet method, with spectra collected from 400 to 4000 cm^−1^ to identify characteristic functional groups. For amino acid composition analysis, Gl-Gp was hydrolyzed in 6 mol/L hydrochloric acid at 110 °C for 24 h, followed by quantification using an automated amino acid analyzer to determine the amino acid profile of the sample.

### 4.5. In Vitro Antioxidant Activity of Gl-Gp

The antioxidant activity of Gl-Gp was evaluated by the DPPH assay, in which 100 μL of 0.1 mmol/L DPPH solution was mixed with 100 μL of Gl-Gp at different concentrations or with VC, incubated in the dark at room temperature, and the absorbance was measured at 517 nm [29]. The ABTS^+^ radical scavenging activity of Gl-Gp was assessed by mixing 200 μL of ABTS solution with 10 μL of Gl-Gp or VC at various concentrations, incubating at room temperature, and measuring absorbance at 734 nm. The total reducing power was assessed by incubating Gl-Gp solutions of different concentrations with PBS and 1% potassium ferricyanide at 50 °C for 20 min, followed by cooling, addition of 10% trichloroacetic acid, and centrifugation. The reducing power of Gl-Gp was measured by reaction with FeCl_3_ and absorbance detection at 700 nm, with VC as a positive control. Anti-lipid peroxidation activity was evaluated by monitoring fluorescence changes in a pyranine-AAPH system (excitation: 456 nm, emission: 512 nm) at 37 °C in the presence of varying Gl-Gp concentrations. All assays were performed in triplicate.

### 4.6. Characterization of Gl-Gp ME

Firstly, the appearance of the microemulsion was visually inspected, followed by centrifugation at 4000 rpm for 15 min at room temperature to assess physical stability by detecting any phase separation or flocculation. The microemulsion type was identified using staining and dilution methods: the staining method involved adding equal volumes of lipophilic Sudan Red and hydrophilic methylene blue dyes to the microemulsion, observing the diffusion behavior of the dyes; the dilution method involved adding isopropyl myristate and distilled water separately to the microemulsion, observing changes in microemulsion state after dilution. Gl-Gp microemulsion morphology was characterized by TEM. Samples were placed on copper grids, negatively stained with 2% phosphotungstic acid, air-dried, and imaged under a tungsten filament TEM to assess droplet structure and size. The pH values of both the blank microemulsion and the Gl-Gp microemulsion were measured using a pH meter. For preliminary stability evaluation, three batches of Gl-Gp microemulsions were sealed in 5 mL EP tubes and stored at room temperature for 10, 20, and 30 days, during which appearance changes were monitored and stability was assessed by measuring encapsulation efficiency and drug loading.

### 4.7. In Vitro Transdermal Permeation Study

The Franz diffusion cell was used with excised abdominal rat skin. The epidermal hair and subcutaneous connective tissue were carefully removed, and the skin was rinsed and dried before mounting on the diffusion cell, with the stratum corneum facing the donor chamber and the dermis facing the receptor chamber, ensuring no air bubbles were present [30]. A total of 1 g of Gl-Gp aqueous solution and Gl-Gp microemulsion were separately added to the donor chamber. Transdermal diffusion of Gl-Gp was assessed at 37 °C with stirring at 3000 rpm. Receptor chamber samples (0.5 mL) were collected at 2, 4, 8, 12, 18, 24, and 36 h, and Gl-Gp concentrations were measured to determine cumulative permeation. The cumulative permeation amount was calculated using the following formula:

$$Q_{n}=\frac{C_{n}V_{0}+\sum_{i=1}^{n-1}C_{i}V_{1}}{∆}$$

where Q_n_ is the cumulative permeation per unit area (mg/cm^2^), C_n_ is the Gl-Gp concentration in the receptor solution at the nth sampling time (mg/mL), V_0_ is the volume of the receptor chamber (mL), C_i_ is the concentration at the ith sampling time, V_1_ is the sampling volume (mL), and A is the effective diffusion area (cm^2^). This method comprehensively evaluates the morphology, stability, and in vitro transdermal performance of the Gl-Gp microemulsion.

### 4.8. Cell Counting Kit 8 (CCK-8) Assay and Calcein Acetoxymethyl Ester (Calcein-AM)/PI Staining

HaCaT cells in the logarithmic growth phase were harvested and seeded into 96-well flat-bottom plates at a density of 1 × 10^3^ cells per well [31]. Logarithmically growing HaCaT cells were seeded in 96-well plates at 1 × 10^3^ cells/well for subsequent viability and cytotoxicity analyses. Following incubation under standard conditions for 24 h, the initial culture medium of the HaCat cells was substituted with varying concentrations (40, 80, or 120 μg/mL) of Gl-Gp solutions and incubated for 24, 48, or 72 h. HaCat cells cultured exclusively in complete medium were assigned to the control group. Cell proliferation was evaluated using a CCK-8 assay, which reflects mitochondrial activity at defined time points following Gl-Gp treatment.

The effects of Gl-Gp on RAW264.7 cell viability were assessed using a calcein-AM/PI double-staining assay following 24, 48, and 72 h of treatment [32]. Cells were incubated with a staining solution containing 2.0 μmol/L calcein-AM and 4.0 μmol/L PI at 37 °C in the dark for 30 min. Subsequently, the cells were immediately subjected to imaging analysis using a Zeiss confocal fluorescence microscope (model HPX-200, Oberkochen, Germany).

### 4.9. In Vitro Hemolysis Assay

For the hemolysis assay, erythrocytes were first isolated from fresh rabbit blood by mixing with an equal volume of phosphate-buffered saline (PBS) and centrifuging at 1000× *g* for 10 min at 4 °C. The collected erythrocytes were washed four times with PBS until the supernatant became clear and subsequently diluted to a 2% suspension at 4 °C. *Ganoderma lucidum* glycoprotein (Gl-Gp) solutions were prepared at concentrations of 40, 80, and 120 μg/mL in PBS and mixed 1:1 with the erythrocyte suspension. The mixtures were incubated at 37 °C for 60 min. PBS and deionized water were employed as negative and positive controls, respectively. After centrifugation at 1000× *g* for 5 min at 20 °C, the supernatants were collected, photographed, and their absorbance measured at 545 nm. Hemolysis ratios (HR) were calculated according to the formula: HR (%) = (Asample - Anegative)/(Apositive - Anegative) × 100%, where A represents the absorbance of each group.

### 4.10. Scratch Migration Assay and Transwell Assay

The migratory capacity of HaCaT cells was evaluated in both two- and three-dimensional models. For the two-dimensional assay, HaCaT cells were seeded in 96-well plates at a density of 2 × 10^4^ cells per well and allowed to form a confluent monolayer. Cells were synchronized in low-serum medium for 12 h before generating a uniform scratch with a 200-μL pipette tip. Detached cells were removed by washing with 0.01 M phosphate-buffered saline (PBS), and Gl-Gp solutions at concentrations of 40, 80, or 120 μg/mL were applied. Cell migration into the wound area was monitored at 0, 12, and 24 h using an inverted microscope, and the scratch closure was quantified using ImageJ software (Version 1.53, National Institutes of Health, USA). In parallel, three-dimensional migratory behavior was assessed via a Transwell assay following Gl-Gp treatment [33]. The migratory potential of HaCaT cells in three-dimensional space was evaluated using a Transwell assay following treatment with Gl-Gp.

A scratch test was used to assess the ability of HaCaT cells to migrate in a two-dimensional plane. Briefly, 96-well plates were seeded with HaCaT cells at a density of 20,000 cells per well to establish a cell monolayer. The cells were synchronized with the addition of low-serum medium for 12 h. Subsequently, the well plates were scored using a 200-μL pipette tip and washed with phosphate-buffered saline (PBS, 0.01 mol/L) to remove damaged cells. Gl-Gp solutions (40, 80, or 120 μg/mL) were subsequently added to the HaCaT cells. Cell migration, in which the cells filled gaps between the edges of the wounds, was monitored using an inverted microscope and photographed at 0, 12, and 24 h. The area of the scratch gap was calculated using the ImageJ software to calculate the migration efficiency.

The three-dimensional migratory behavior of HaCaT cells was assessed using a Transwell assay following Gl-Gp treatment [34]. Cells were serum-starved for 24 h, digested with trypsin, and resuspended at a concentration of 8 × 10^5^ cells/mL. A total of 100 μL of the cell suspension, together with the corresponding Gl-Gp solution, was added to the upper chamber, while 600 μL of DMEM containing 10% FBS was placed in the lower chamber. After 48 h of incubation in a CO_2_ incubator, migrated cells on the lower surface of the membrane were fixed with methanol, stained with 0.1% crystal violet, and washed three times with water. Non-migrated cells on the upper surface were gently removed with a cotton swab, and images were captured under a microscope at 100× magnification.

### 4.11. UVB Irradiation and Gl-Gp Treatment on HaCaT Cells

HaCaT cells were seeded in 96-well plates at 1 × 10^4^ cells/well and cultured with Gl-Gp-containing medium [35]. At ~80% confluence, cells were synchronized in low-glucose medium with Gl-Gp for 12 h. Prior to UVB exposure, a thin layer of PBS (0.01 mol/L) was added to prevent desiccation. Cells were irradiated at 140 mW/cm^2^ for 360 s (cumulative dose 50 mJ/cm^2^) with a 30 cm lamp distance. Post-irradiation, PBS was removed and cells were incubated with Gl-Gp for 24 h. Proliferation and viability were assessed using CCK-8 and calcein-AM/PI staining [36].

### 4.12. 2,7-Dichlorodi-Hydrofluorescein Diacetate (DCFH-DA) Staining

ROS levels in HaCaT cells were assessed using DCFH-DA staining [37]. Cells were seeded at 1 × 10^4^ cells/well and pretreated with varying concentrations of Gl-Gp for 24 h, followed by UVB irradiation [38]. DCFH-DA was diluted 1:1000 in serum-free medium and applied to the cells for 20 min at 37 °C. Excess probe was removed by three washes with serum-free medium before fluorescence measurement.

### 4.13. Flow Cytometric Analysis of Apoptosis

HaCaT cells were plated in six-well plates at 5 × 10^5^ cells/well and pretreated with various concentrations of Gl-Gp for 24 h. Following UVB exposure, cells were trypsinized, collected, and centrifuged at 1000× *g* for 5 min to obtain a single-cell suspension. Apoptosis was evaluated using an Annexin V-FITC/PI kit (Beyotime Biotechnology, Shanghai, China, Cat. No. C1062). Cells were incubated with Annexin V-FITC and PI for 15 min in the dark, resuspended in 400 μL of binding buffer, and maintained on ice prior to flow cytometric analysis to quantify early and late apoptotic populations.

### 4.14. JC-1 Staining and Flow Cytometric Analysis of Mitochondrial Membrane Potential

Mitochondrial membrane potential (ΔΨm) was evaluated using JC-1 staining combined with flow cytometry [39]. HaCaT cells were seeded into 6-well plates at a density of 1 × 10^6^ cells per well and incubated for 24 h. HaCaT cells were seeded in six-well plates at 1 × 10^6^ cells/well and incubated for 24 h. Cells were treated with different concentrations of Gl-Gp and subjected to UVB irradiation, followed by an additional 6 h of Gl-Gp exposure [40]. Cells were harvested with EDTA-free trypsin, washed twice with cold PBS, and incubated with JC-1 working solution (10 µg/mL) at 37 °C for 20 min in the dark. After centrifugation at 600× *g* for 5 min and a wash with JC-1 staining buffer, cells were analyzed by flow cytometry. JC-1 monomers (green, FITC channel) and aggregates (red, PE channel) were quantified, and the red/green fluorescence ratio was calculated to determine ΔΨm changes.

### 4.15. Photodamage Model in Murine Skin

After anesthesia with isoflurane, the dorsal skin of each rat was shaved, and a 4 × 4 cm^2^ area was depilated using a commercial hair removal cream. Sprague-Dawley rats were randomly assigned to six groups: control (no UVA + UVB exposure), UVA + UVB model, low- and high-dose Gl-Gp treatment (40 and 120 mg/kg, respectively), Gl-Gp microemulsion (Gl-Gp ME), and positive control (vitamin E, VE) [41]. UVA + UVB irradiation was delivered using parallel lamps positioned 15 cm above the dorsal area, with a daily dose of 200 mJ/cm^2^ for 21 consecutive days, resulting in a cumulative dose of 1.4 J/cm^2^.

### 4.16. Pathological Histopathological Analysis

Dorsal skin photographs were captured throughout the treatment period, and skin thickness at the same site was measured using a vernier caliper to monitor recovery [42]. Assays for MDA, SOD, GSH-Px, HYP, MMP-1, MMP-3, IL-6, IL-1β, and TNF-α were performed according to the respective kit protocols [43].

Skin tissues were fixed in 4% paraformaldehyde for 24 h, rinsed with running water for 6 h, and dehydrated through a graded ethanol series (50%, 75%, 85%, 95%, 100%) [44]. Tissues were cleared with xylene, embedded in paraffin, and sectioned at ~4 μm. Histological evaluation of pathological changes and collagen fiber deposition was performed using H&E and aldehyde fuchsin staining, and the sections were digitally scanned for analysis.

### 4.17. Confocal Fluorescence Microscopy for Evaluation of Skin Permeation of Gl-Gp Microemulsion

To evaluate the transdermal penetration and distribution of the Gl-Gp microemulsion (Gl-Gp ME) in skin tissue, the lipid phase of the formulation was labeled with the lipophilic fluorescent tracer PKH67 by co-incubation at 4 °C for 12 h under dark conditions. The labeled Gl-Gp ME was then evenly applied to the depilated dorsal skin of rats at predetermined doses. After 6 h of topical application, the animals were euthanized, and the treated skin tissues were collected, embedded in O.C.T. compound, and sectioned longitudinally at a thickness of approximately 10 μm using a cryostat. The frozen sections were washed three times with PBS (pH 7.4, 5 min each), followed by incubation with DAPI staining solution for 10 min at room temperature in the dark. After rinsing, the sections were mounted with an anti-fluorescence quenching mounting medium. Fluorescence images were acquired using a confocal laser scanning microscope (Leica TCS SP8, Leica Microsystems, Wetzlar, Germany) to detect DAPI (Ex 330–380 nm/Em 420 nm) and PKH67 (Ex 465–495 nm/Em 515–555 nm) signals. in order to visualize the distribution of the formulation across the epidermis, dermis, and subcutaneous layers.

## 5. Conclusions

In this study, *Ganoderma lucidum* glycoprotein (Gl-Gp) was successfully extracted and structurally characterized as a typical O-glycosylated glycoprotein featuring abundant O-GlcNAc modifications, pyranose-type polysaccharide chains, and a diverse amino acid composition. These structural attributes confer potent antioxidant capacity, as verified by multiple in vitro assays, including DPPH and ABTS^+^ radical scavenging, total reducing power, and lipid peroxidation inhibition. Gl-Gp exhibited excellent biocompatibility, showing no cytotoxicity toward HaCaT and RAW264.7 cells, negligible hemolytic activity, and a strong ability to promote cell proliferation and migration. Mechanistic investigations demonstrated that Gl-Gp protects UVB-exposed cells by scavenging intracellular reactive oxygen species (ROS), maintaining mitochondrial membrane potential, and reducing apoptosis.

Furthermore, a water-in-oil (W/O) microemulsion (Gl-Gp ME) was developed to enhance transdermal delivery, significantly improving percutaneous permeation and formulation stability. In vivo experiments using a UVA + UVB-induced photodamaged rat model revealed that Gl-Gp, particularly in high-dose and microemulsion formulations, effectively alleviated erythema, scabbing, skin dehydration, and structural disruption. Histological and biochemical analyses showed that Gl-Gp mitigated epidermal hyperplasia, restored the organization of collagen and elastic fibers, reduced MDA accumulation, increased SOD, GSH-Px, and HYP levels, and downregulated the expression of MMP-1, MMP-3, IL-1β, IL-6, and TNF-α. Collectively, these findings demonstrate that Gl-Gp exerts multi-targeted protective effects against photoaging through antioxidant, anti-inflammatory, and extracellular matrix-modulating mechanisms, while the microemulsion formulation further enhances efficacy by facilitating transdermal absorption.

Although fluorescence imaging provided preliminary evidence that Gl-Gp ME could penetrate into the viable epidermis and partially into the dermis, further studies using more physiologically relevant skin models are required to verify the underlying mechanisms, particularly those related to mitochondrial protection. Future research will focus on elucidating the in-skin mechanisms of Gl-Gp and confirming its antioxidant and mitochondrial regulatory effects in viable human or animal skin systems.

It should also be noted that the composition of *Ganoderma lucidum* glycoproteins may vary with strain and cultivation conditions, which could influence their biological activity. Therefore, future comparative analyses of glycoproteins derived from different sources will be conducted to evaluate the reproducibility and generalizability of these findings.

In addition, we will further systematically investigate the intradermal pharmacokinetic characteristics, tissue distribution, and metabolic fate of Gl–Gp to clarify its retention and clearance behavior within different skin layers and to elucidate the kinetic basis underlying its sustained antioxidant and tissue-repairing effects. On this basis, we plan to integrate advanced drug delivery strategies, such as microneedle-assisted administration, hybrid nanocarriers, or intelligent thermo-responsive systems, to further improve the dermal targeting efficiency and bioavailability of Gl–Gp. Moreover, comprehensive assessments under real-use conditions will be conducted to evaluate the long-term stability, skin tolerance, and potential irritation of the formulation, and the results will be used to optimize the formulation system and application protocol. By deepening the mechanistic investigations and actively advancing clinical validation, this work provides a solid scientific foundation for translating the Gl–Gp microemulsion from the laboratory stage to clinical application.

However, we also acknowledge several important limitations of the present study. The current findings were mainly derived from in vitro models and a UV-induced photoaging rat model, which do not fully reproduce the complex structural and physiological characteristics of human skin. Differences between rodents and humans in stratum corneum thickness, lipid composition, and vascular distribution may lead to variations in transdermal behavior and pharmacodynamic outcomes. Although this study confirmed the dermal delivery, antioxidant, and anti-inflammatory activities of Gl–Gp, the intradermal pharmacokinetics, metabolic fate, and long-term topical safety have not yet been quantitatively characterized. While no irritation was observed in animal experiments, human skin responses may differ, particularly in individuals with sensitive skin or impaired barrier function. More critically, the absence of clinical data limits the immediate translational applicability of our findings. Therefore, we plan to conduct a randomized, double-blind, split-face clinical study in volunteers with mild to moderate photoaging, in which the Gl–Gp microemulsion will be applied to one side of the face and the placebo vehicle to the other for 28 consecutive days. Before and after treatment, non-invasive instrumental assessments—including the cutometer, mexameter, corneometer, and TEWL measurements—will be performed to objectively evaluate skin elasticity, firmness, pigmentation, and barrier function, thereby providing a more comprehensive understanding of the safety and preliminary efficacy of the Gl–Gp formulation.

## Data Availability

The original contributions presented in this study are included in the article. Further inquiries can be directed to the corresponding authors.

## Abbreviations

The following abbreviations are used in this manuscript:

Gl-Gp	*Ganoderma lucidum* glycoprotein
Gl-Gp ME	*Ganoderma lucidum* glycoprotein microemulsion
